# BDNF Binds Its Pro-Peptide with High Affinity and the Common Val66Met Polymorphism Attenuates the Interaction

**DOI:** 10.3390/ijms18051042

**Published:** 2017-05-12

**Authors:** Koichi Uegaki, Haruko Kumanogoh, Toshiyuki Mizui, Takatsugu Hirokawa, Yasuyuki Ishikawa, Masami Kojima

**Affiliations:** 1 Biomedical Research Institute (BMD), National Institute of Advanced Industrial Science and Technology (AIST), 1-8-31 Midorioka, Ikeda, Osaka 563-8577, Japan; k-uegaki@aist.go.jp (K.U.); h.kumanogoh@gmail.com (H.K.); t-mizui@aist.go.jp (T.M.); 2 Core Research for Evolutional Science and Technology (CREST), Science and Technology Agency (JST), Kawaguchi, Saitama 332-0012, Japan; t-hirokawa@aist.go.jp; 3 Molecular Profiling Research Center for Drug Discovery, National Institute of Advanced Industrial Science and Technology (AIST), Tokyo 135-0064, Japan; 4 Division of Biomedical Science, Faculty of Medicine, University of Tsukuba, 1-1-1 Tennodai, Tsukuba-shi, Ibaraki 305-8575, Japan; 5 Department of Systems Life Engineering, Maebashi Institute of Technology 460-1, Kamisadori, Maebashi 370-0816, Japan; 6 Graduate School of Frontier Bioscience, Osaka University, Suita 565-0871, Japan

**Keywords:** BDNF, pro-peptide, polymorphism, long-term depression, hippocampus

## Abstract

Most growth factors are initially synthesized as precursors then cleaved into bioactive mature domains and pro-domains, but the biological roles of pro-domains are poorly understood. In the present study, we investigated the pro-domain (or pro-peptide) of brain-derived neurotrophic factor (BDNF), which promotes neuronal survival, differentiation and synaptic plasticity. The BDNF pro-peptide is a post-processing product of the precursor BDNF. Using surface plasmon resonance and biochemical experiments, we first demonstrated that the BDNF pro-peptide binds to mature BDNF with high affinity, but not other neurotrophins. This interaction was more enhanced at acidic pH than at neutral pH, suggesting that the binding is significant in intracellular compartments such as trafficking vesicles rather than the extracellular space. The common Val66Met BDNF polymorphism results in a valine instead of a methionine in the pro-domain, which affects human brain functions and the activity-dependent secretion of BDNF. We investigated the influence of this variation on the interaction between BDNF and the pro-peptide. Interestingly, the Val66Met polymorphism stabilized the heterodimeric complex of BDNF and its pro-peptide. Furthermore, compared with the Val-containing pro-peptide, the complex with the Met-type pro-peptide was more stable at both acidic and neutral pH, suggesting that the Val66Met BDNF polymorphism forms a more stable complex. A computational modeling provided an interpretation to the role of the Val66Met mutation in the interaction of BDNF and its pro-peptide. Lastly, we performed electrophysiological experiments, which indicated that the BDNF pro-peptide, when pre-incubated with BDNF, attenuated the ability of BDNF to inhibit hippocampal long-term depression (LTD), suggesting a possibility that the BDNF pro-peptide may interact directly with BDNF and thereby inhibit its availability. It was previously reported that the BDNF pro-domain exerts a chaperone-like function and assists the folding of the BDNF protein. However, our results suggest a new role for the BDNF pro-domain (or pro-peptide) following proteolytic cleave of precursor BDNF, and provide insight into the Val66Met polymorphism.

## 1. Introduction

Growth factors control many cellular functions including proliferation, differentiation and cell migration. In the nervous system, brain-derived neurotrophic factor (BDNF) [1,2] belongs to the neurotrophin family along with proteins such as nerve growth factor (NGF), neurotrophin-3 (NT-3) and neurotrophin-4 (NT-4) [3]. One biological action of BDNF is the promotion of differentiation and survival of neurons [1,4]. Interestingly, it was reported that BDNF was highly expressed in the brain and that expression was regulated at transcriptional levels by neuronal activity [5]. BDNF has been shown to be crucial in developing and controlling synapses with functional and structural effects [6,7,8]. It is now widely accepted that in the adult brain the central function of BDNF is to control structure and function of synapses ranging from short-term to long-term and on excitatory and inhibitory neurons, in many brain regions [8,9,10].

**Figure 1:**
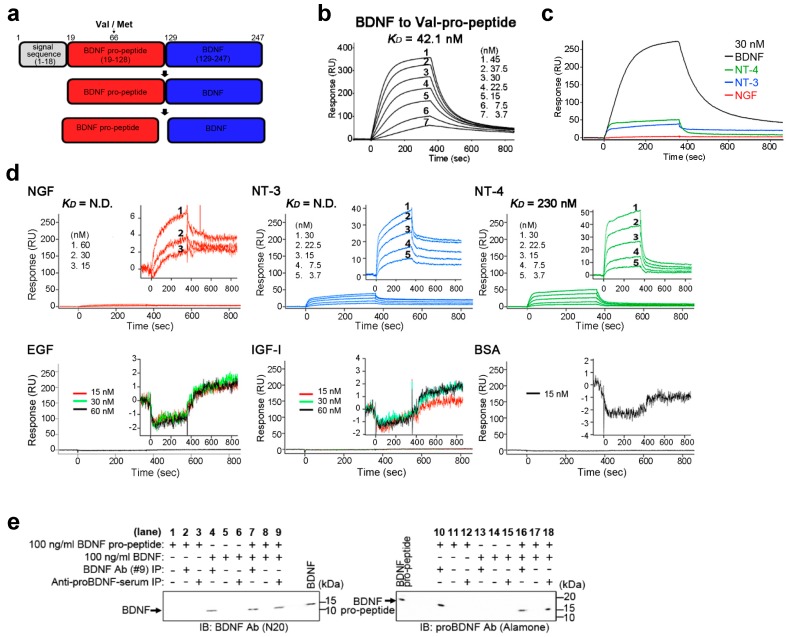
BDNF binds its pro-peptide with high affinity. (**a**) Schematic representation of the BDNF precursor (pro-BDNF), BDNF and its pro-peptide. The number of amino acids in the signal sequence, the BDNF pro-peptide (BDNF pro-domain), and the mature BDNF is shown. The small black arrow designates the location of the Val66Met mutation; (**b**) Binding curves for BDNF and the BDNF pro-peptide. BIAcore binding assays were performed as described in the Materials and Methods. Briefly, recombinant wild-type BDNF pro-peptide (Val in Figure 1a) was immobilized on the chip, and surface plasmon resonance (SPR) response curves were plotted against the indicated concentration (nM) of BDNF. RU, resonance units. *K*_D_, dissociation constant; (**c**) Binding of BDNF pro-peptide to BDNF. Val-BDNF pro-peptide was immobilized on the BIAcore sensor chip. SPR response curves are plotted for the indicated neurotrophic factors at 30 nM. RU, resonance units; (**d**) Other neurotrophins and growth factors dose not bind the BDNF pro-peptide. In the BIAcore assays, Val-BDNF pro-peptide was immobilized on the BIAcore sensor chip. In all graphs, SPR response curves are plotted for the growth factors and protein concentrations indicated on each graph. *K*_D_ values are also shown. RU, resonance units. Inset graphs magnify the *y*-axis. N.D., not detectable. Note that compared to BDNF, the growth factors tested here exhibited no or weak binding to the BDNF pro-peptide, and that despite significant amino-acid sequence similarity to BDNF, other neurotrophins and growth factors did not bind the BDNF pro-peptide. Bovine serum albumin (BSA) was used as a negative control; (**e**) In vitro binding of BDNF with its pro-peptide. BDNF and/or the pro-peptide were incubated for 1 h then subjected to immunoprecipitation with the indicated antibodies. Binding of BDNF and its pro-peptide was analyzed by western blotting with the indicated antibodies.

As with other growth factors, BDNF is synthesized as a precursor protein (proBDNF) [2]. An approximate 100 amino acid N-terminal pro-domain (pro-peptide) is cleaved from proBDNF to produce the biologically active BDNF protein (Figure 1a). The BDNF pro-domain reportedly acts as a molecular chaperone to assist the folding of the BDNF protein [11], and recent reports suggested additional roles for this domain. It was demonstrated that the immunoreactive signal of the BDNF pro-peptide is present in the presynaptic vesicles of brain neurons [12], suggesting the BDNF pro-peptide itself is carried and released in an intracellular vesicle-dependent manner. Furthermore, the BDNF pro-peptide is endogenously releasable and controls the cellular mechanisms of axon growth and functional and structural plasticity [13,14,15]. In addition, the single nucleotide polymorphism Val66Met (Figure 1a, arrowhead), in which valine is replaced by methionine at residue 66 in the pro-peptide region of human BDNF, affects brain functions and the activity-dependent secretion of BDNF [16], and other research links the Val66Met mutation with brain functions in human and model mouse [17,18,19]. Thus, the BDNF pro-domain appears to have a number of roles in the nervous system.

The primary sequence of the BDNF pro-peptide is conserved among species and differs significantly from that of other neurotrophins [20,21]. BDNF and its pro-peptide are basic and acidic, respectively, with an isoelectric point (pI) of 9.6 and 5.2 [1,2], which suggests they may interact in an electrostatic manner. Thus, in the present study, we tested this putative interaction using surface plasmon resonance and biochemical methods. The results confirmed binding between BDNF and its pro-peptide, but not between neurotrophins NGF, NT-3 and NT-4, which share significant amino acid sequence similarity with BDNF [3,22]. Furthermore, the Val66Met polymorphism stabilized the interaction. The interaction between BDNF and the Met-type pro-peptide was less sensitive to pH changes than the Val-type pro-peptide. These findings indicate novel roles for the BDNF pro-domain and provide new insight into the common Val66Met BDNF polymorphism.

## 2. Results

### 2.1. BDNF Binds Its Pro-Peptide with High Affinity

To examine whether BDNF binds its pro-peptide (Figure 1a), we immobilized recombinant wild-type (WT) BDNF pro-peptide (Val in Figure 1a) on a BIAcore sensor chip and measured the binding to BDNF. Administration of BDNF led to a rapid and reversible response on the BIAcore sensor chip, and binding and release occurred in a BDNF concentration-dependent manner (Figure 1b, 3.7–45 nM). The dissociation constant *K*_D_ (concentration at half-maximal binding) was calculated to be 42.1 nM (Figure 1b), indicating high affinity binding between BDNF and its pro-peptide. To confirm the specificity of binding, we tested NGF, NT-3 and NT-4, which are significant amino acid sequence similarity with BDNF, but none showed significant binding to the BDNF pro-peptide (Figure 1c; NGF, NT-3 and NT-4. This also held true for more detailed experiments with NGF, NT-3 and NT-4 at different concentrations (Figure 1d; NGF, NT-3 and NT-4). Moreover, epidermal growth factor (EGF), insulin-like growth factor 1 (IGF-1) and bovine serum albumin (BSA) showed no binding to the BDNF pro-peptide (Figure 1d; EGF, IGF-1 and BSA). The surface plasmon resonance data were further supported by biochemical experiments. Notably, a 1 h pre-incubation of BDNF with its pro-peptide resulted in the precipitation of the BDNF/pro-peptide complex (Figure 1e, lanes 9 and 16), but other protein-peptide complexes were not precipitated. Thus, these results suggest that BDNF pro-peptide binds to BDNF with high affinity and specificity.

### 2.2. The BDNF Val66Met Polymorphism Stabilizes Interaction with Its Pro-Peptide

**Figure 2:**
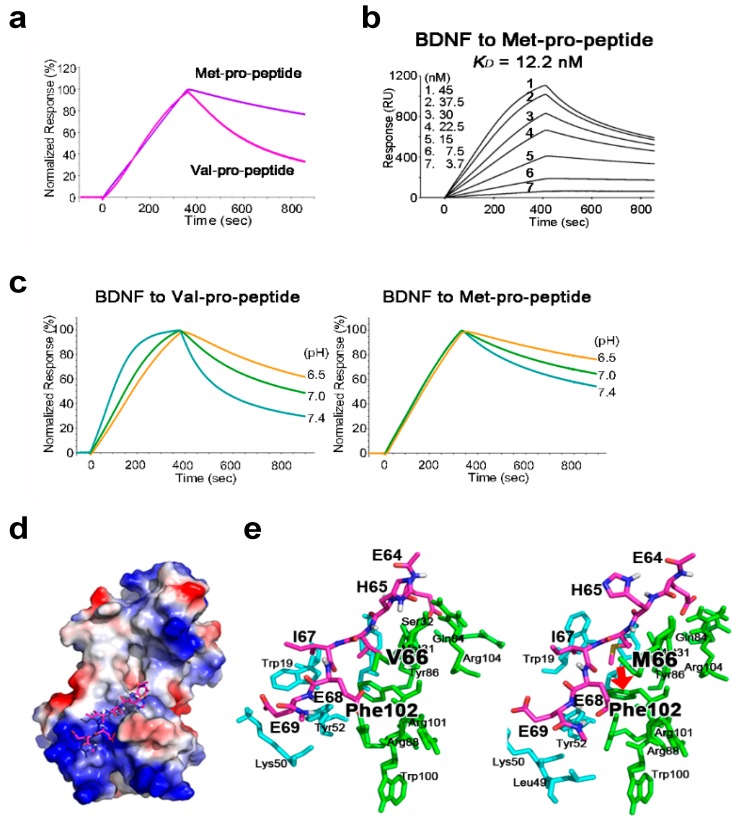
Impact of the Val66Met mutation on the biological role of the BDNF pro-peptide. (**a**) Normalized binding curves for BDNF and the BDNF pro-peptide with Val or Met at position 66 were determined using BIAcore assays. The recombinant BDNF pro-peptide with Val or Met was immobilized on the chip and the BIAcore binding assay was performed. The concentration of BDNF used in this figure was 30 nM; (**b**) Binding curves for BDNF and the Met-BDNF pro-peptide. In graphs, SPR response curves are plotted against the indicated concentration of BDNF and the dissociation constant, *K*_D_. RU, resonance unit; (**c**) Impact of the Val66Met polymorphism on the pH-dependent release of BDNF from its pro-peptide. In left and right, SPR responses are normalized against the response obtained when BDNF was washed off. The concentration of BDNF used was 30 nM. Note that the Met variant pro-peptide dissociates from BDNF more slowly than the wild-type (Val) pro-peptide at all pH values tested; (**d**) Docking simulations between short fragments encompassing Val or Met at position 66 and the mature BDNF protein. An overview of a model of the binding of the six amino acid residue peptide flanking valine 66 (stick representation) is shown in front of the BDNF structure. The electrostatic surface of BDNF was created using PyMOL software (Schrödinger, LLC, New York, NY, USA); (**e**) Close-up view of the modeled interaction between BDNF and the six amino acid residue peptide with valine (left) or methionine (right). BDNF residues surrounding the BDNF pro-peptide within 4 Å are shown in stick representation. Note that the Cε atom of methionine 66 engages in π-δ interactions with phenylalanine at position 102 in BDNF (right, red arrow).

A body of evidence indicates that the BDNF Val66Met polymorphism affects human brain function [16,23]. Since the Val66Met polymorphism substitutes valine for methionine at position 66 in the BDNF pro-peptide, we examined whether this polymorphism affected the interaction between BDNF and its pro-peptide using recombinant BDNF pro-peptide with Val or Met immobilized on a BIAcore chip as shown in Figure 1b. Interestingly, compared with the Val-BDNF-pro-peptide, the Met-BDNF-pro-peptide apparently released BDNF protein very slowly (Figure 2a, Val/Met pro-peptide concentration = 30 nM) and in a BDNF concentration-dependent manner (Figure 2b, 3.7–45 nM). Quantitative analysis revealed that the Val66Met mutation decreased the rate of association (*k*_a_) and dissociation (*k*_d_) by 10- and 100-fold, respectively (Table 1, *k*_a_ and *k*_d_). These results suggest the Val66Met polymorphism stabilizes the molecular interaction between BDNF and its pro-peptide.

### 2.3. The Met-Containing BDNF Pro-Peptide Binds Stably to BDNF over A Wider pH Range

BDNF is transported from acidic intracellular organelles to the neutral extracellular space [9]. The Val66Met BDNF polymorphism reportedly affects the intracellular trafficking of BDNF [16,23]; therefore, we raised the question of whether binding of BDNF to its pro-peptide is sensitive to pH. To test this, we compared binding of BDNF to Val- and Met-containing pro-peptide at pH 6.5–7.4 using a BIAcore chip. Interestingly, at all conditions tested, the Met-BDNF pro-peptide released BDNF more slowly than did the Val-type pro-peptide, and the association and dissociation with BDNF appeared to be independent of pH. (Figure 2c; Val- and Met-BDNF pro-peptide, 30 nM). These results suggest that the Met mutation enhances the stability of binding in the BDNF/pro-peptide complex over a wider pH range (pH 6.5–7.4), providing a new explanation for the molecular role of the BDNF Val66Met polymorphism.

### 2.4. Structural Analysis of BDNF/Pro-Peptide Interaction and the Influence of the Val66Met Polymorphism

To interpret the role of the Val66Met polymorphism in the BDNF/pro-peptide complex, performed a docking simulation with a six amino acid pro-peptide fragment encompassing Val or Met at position 66 (Figure 2d, stick representation). In this simulation, the short BDNF pro-peptide fragment appeared to interact with basic electrostatic patches formed by five amino acid residues (K10, R88, F102, R101 and R104) in the BDNF protein. When valine 66 was replaced by methionine, the methyl group of the substituted residue forms a new and strong hydrophobic interaction with the aromatic side chain of phenylalanine at position 102 in BDNF (Figure 2d; right, indicated by the bold arrow), whereas the shorter side chain of valine in the WT pro-peptide does not form such a significant interaction (Figure 2d, left). Together, our combined biochemical and computational modeling results suggest a novel role for the BDNF pro-peptide, and provide insight into the molecular role of the common BDNF Val66Met polymorphism involving an amino acid substitution in the pro-peptide.

### 2.5. Interaction of BDNF and Its Pro-Peptide May Affect the Biological Function of BDNF

**Figure 3:**
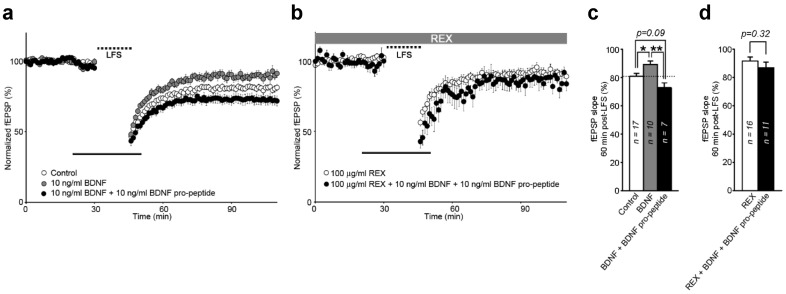
Interaction of BDNF and its pro-peptide may affect the biological function of BDNF. LTD was induced by low-frequency stimulation (LFS) (1 Hz; 900 pulses) to the Schaeffer collaterals along with treatment using the indicated drug. In graphs, symbols represent the mean values of the field excitatory postsynaptic potential (fEPSP) slope. The 100% value corresponds to the pre-LFS baseline. The summary histogram depicts LTD measured 60 min after LFS application. The number of slices used is associated with the bar graph. * *p* < 0.05; ANOVA with post-hoc test. (**a**) Attenuation in the activity of BDNF by the BDNF pro-peptide modulates plasticity. The wild-type BDNF pro-peptide was pre-incubated with or without BDNF for 1 h and applied to hippocampal slices. Electrophysiology was performed as detailed in the Materials and Methods; (**b**) REX (100 μg/mL) shows no effect in electrophysiological experiments performed as described in (**a**); (**c**,**d**) LTD measured 60 min after LFS stimulation. (**c**,**d**) represent data from (**a**,**d**), respectively.

Although electrostatic protein-protein interactions operate over a relatively long range, they can have a large influence on biological events [30]. Dieni et al. (2012) showed that BDNF and its pro-peptide are endogenously stored in presynaptic dense-core vesicles in excitatory presynaptic terminals in the adult mouse brain [12], indicating their interaction after co-release. Thus, we reasoned that the interaction between BDNF and its pro-peptide may affect the biological function of BDNF and/or its pro-peptide. We performed electrophysiological experiments (Figure 3), which indicated that the BDNF pro-peptide, when pre-incubated with BDNF, attenuated the ability of BDNF to inhibit hippocampal long-term depression (LTD) (Figure 3a,c). Furthermore, this attenuation was not rescued by treatment with REX, a polyclonal antibody against the rat extracellular domain of p75^NTR^ (Figure 3b,d). Recently, we reported that the BDNF pro-peptide facilitates hippocampal LTD, and this enhancement requires p75^NTR^, while BDNF successfully rescued hippocampal LTD [15]. Thus, these data suggest a possibility that the BDNF pro-peptide may interact directly with BDNF and thereby inhibit its availability.

## 3. Discussion

Post-translational mechanisms of growth factors are known to diversify and specify the functions of proteins [24]. However, compared with accrued knowledge of the expression and functions of growth factors, much less is known about the role of post-translational mechanisms. In the present study, we focused on the BDNF pro-domain (pro-peptide), which is generated by proteolytic processing of the precursor BDNF (Figure 1a). Firstly, we demonstrated that BDNF binds its pro-peptide with high affinity, while other neurotrophins showed an extremely weak binding to the BDNF pro-peptide. Secondly, we showed that the interaction of BDNF and its pro-peptide was enhanced in the common BDNF Val66Met polymorphism, in which valine is replaced by a methionine in the pro-peptide (Figure 1a). Thirdly, we found that the interaction of BDNF and its pro-peptide was more stable in acidic rather than neutral pH conditions. Interestingly, the Val66Met polymorphism increased the stability of binding across the entire acidic to neutral pH range. These results suggest a new role for the BDNF pro-peptide that is generated by processing of the proBDNF form, and offer insight into the effects of the common BDNF Val66Met polymorphism.

The mature BDNF domain is highly conserved among vertebrates [2,25], including in the primary sequence of the BDNF pro-region of human and chicken proteins [21,26]. Thus, it is believed that the pro-region/pro-peptide of BDNF may exert not only a structural role during protein folding [11], but also specific biological functions [27]. In addition, BDNF and its pro-peptide are basic and acid, respectively, with an iso-electric point (pI) of 9.6 and 5.2 [1], raising the possibility that these proteins interact electrostatically, and that the pro-peptide may affect the availability of BDNF and, hence, its bioactivity.

To test this, we examined the binding of BDNF and its pro-peptide using surface plasmon resonance with BIAcore binding assays. The binding affinity of BDNF for its pro-peptide was approximately 42.1 nM (*K*_D_ indicated in Figure 1b). Furthermore, this interaction was specific because the BDNF pro-peptide did not show an apparent interaction with other neurotrophins (NGF, NT-3 and NT-4) sharing significant homology with BDNF [3]. In contrast, it was previously reported that there was no significant binding between NGF and its pro-peptide [28], suggesting that although the mature domains of NGF and BDNF are homologous in terms of amino acid sequence, their pro-domains may exert distinct roles and display functional heterogeneity.

The interaction between BDNF and its pro-peptide was affected at the molecular level. The BDNF Val66Met polymorphism, in which a valine is replaced with a methionine in the pro-peptide, is associated with brain diseases [17,18,19]. In the present study, we observed that this polymorphism pronouncedly influenced the binding kinetics of BDNF to its pro-peptide on the BIAcore chip (Figure 2) and the BDNF/Met-pro-peptide complex dissociated more slowly than the BDNF/Val-pro-peptide complex. Furthermore, quantitative analysis revealed that the Met-containing pro-peptide exhibited decreased rates of association (*k*_a_) and dissociation (*k*_d_) by 10- and 100-fold, respectively. To better understand the role of the Val66Met polymorphism in the interaction of BDNF and its pro-peptide, we performed a computational docking simulation using a six amino acid BDNF pro-peptide fragment encompassing Val or Met at position 66 (Figure 2d,e). Interestingly, strong π–δ hydrophobic interactions between the methyl group of methionine at position 66 and the aromatic side chain of phenylalanine at position 102 were observed in our docking simulation (Figure 2e, red arrow). Therefore, these computational docking results support those of the surface plasmon resonance experiments (Figure 2a,b).

Most growth factors are transported from acidic intracellular vesicles and released into the neutral extracellular space [9]. As shown in Figure 2, the interaction between BDNF and the WT (Val) pro-peptide was sensitive to pH change (pH 6.5, 7.0 and 7.4). At lower pH, the association and dissociation of the complex were slow relative to those under neutral conditions. More interestingly, the mutant Met-pro-peptide released BDNF more slowly than the WT Val-pro-peptide at all pH conditions tested, suggesting the Val66Met polymorphism augments the stability of the BDNF/pro-peptide complex and delays their dissociation.

Although there is a body of evidence speculating on the impact of the Val66Met polymorphism in human brain functions and diseases, the role of this variation at the molecular level has not been fully elucidated. Previously, it was reported that the BDNF pro-domain assists the folding of BDNF by acting as a molecular chaperone [11]. We previously reported that the BDNF Val66Met polymorphism impairs intracellular trafficking of BDNF [16]. In this previous report, we demonstrated that the Val66Met mutation did not affect the efficiency of proBDNF processing in culture [16]. Thus, given this data [16], the BDNF Val66Met polymorphism might contribute to the stability of the BDNF/its pro-peptide complex rather than the mechanism of proBDNF processing. Additionally, the Vps10p protein sortilin reportedly interacts with the BDNF pro-domain, and the Val66Met mutation increases the interaction of the BDNF pro-region with BDNF rather than with sortilin, leading to impairment in intracellular trafficking and secretion of BDNF [29]. Thus, the results of the present study expand our knowledge of the molecular role of the BDNF pro-peptide and the impact of the BDNF Val66Met polymorphism.

Electrostatic protein-protein interactions operate over a relatively long range, they can have a large influence on biological events [30]. Recently, it was reported that BDNF and its pro-peptide are endogenously stored in presynaptic dense-core vesicles in excitatory presynaptic terminals in the adult mouse brain [12], suggesting their interaction after co-release. In the present study, we showed that the BDNF pro-peptide, when pre-incubated with BDNF, attenuated the ability of BDNF to inhibit hippocampal LTD and this inhibition was not rescued by treatment with a REX p75^NTR^ antagonist. Recently, we reported that the BDNF pro-peptide facilitates hippocampal LTD, and this enhancement requires p75^NTR^, while BDNF successfully rescued hippocampal LTD [15]. Taken together with our recent report [15], the electrophysiological data shown in Figure 3 raise a possibility that the BDNF pro-peptide may interact directly with BDNF and thereby inhibit its availability. When present and functioning independently, both proteins (BDNF and its pro-peptide) could engage in their own separate biological activities. We also reported that the BDNF pro-peptide containing the Met mutation could rescue hippocampal LTD [15]. Thus, it will be interesting in future to investigate whether BDNF and its pro-peptide act together to control neuronal functions, and to study how the BDNF Val66Met polymorphism modulates the functional balance of these proteins.

## 4. Materials and Methods

### 4.1. Reagents

Recombinant human BDNF was kindly provided by Sumitomo Pharmaceuticals (Osaka, Japan). Monoclonal anti-BDNF antibody (mAb#9) was kindly provided by Yves-Alain Barde (Cardiff University, Cardiff, UK). The p75 NTR-blocking REX antibody was kindly provided by Louis French Reichardt (University of California, San Francisco, CA, USA). Recombinant human nerve growth factor (NGF) and Bovine serum albumin (BSA) were from Sigma (St. Louis, MO, USA). Recombinant human neurotrophin-3 (NT-3) was from Alomone Labs (Jerusalem, Israel). Recombinant human neurotrophin-4 (NT-4). Recombinant human epidermal growth factor (EGF) was from Biomedical Technologies (Stoughton, MA, USA). Recombinant human insulin-like growth factor I (IGF-I) was from PeproTech (Rocky Hill, NJ, USA). Polyclonal anti-BDNF (pAbN20) was purchased from Santa Cruz Biotechnology (Dallas, CA, USA). Polyclonal anti-proBDNF antibody was purchased from Alomone Labs (batch AN-03, Jerusalem, Israel). Rabbit proBDNF antisera were generated in our laboratory [31].

### 4.2. Production of Recombinant Proteins

Expression plasmids for the BDNF pro-peptide (residues 19–128 of human precursor BDNF), without or with the Met amino-acid substitution at position 66 were generated as described in our previous report [15]. A His-tag and PreScission Protease recognition sequence (LEVLFQGP) was added to the N-terminus of pro-peptide. The resulting constructs were subcloned into the pET-19b vector (Novagen, Madison, WI, USA) and bi-directionally sequenced to confirm the amino-acid sequence. Constructs were expressed in *E. coli* strain BL21 (DE3) pLysS (Novagen, Madison, WI, USA) and purified by liquid chromatography as in our previous report [32]. The molecular weights of the purified proteins were confirmed using matrix-assisted laser-desorption ionization time-of-flight mass spectrometry (Applied Biosystems, Foster City, CA, USA) and were identical to the expected values calculated from the amino-acid sequences.

### 4.3. BIAcore Assay

SPR assays were carried out on a BIAcore 2000 system (GE Healthcare, Little Chalfont, UK) as described in our previous report [15]. Briefly, the BDNF pro-peptide without or with the Met mutation was immobilized on a CM5 research-grade sensor chip (GE Healthcare). Coupling at a density of approximately 2000 RU was achieved with an amine coupling kit (GE Healthcare). A 1:1 mixture of 1-ethyl-3-3-dimethylaminopropyl carbodiimide hydrochloride and *N*-hydrosuccinimide was used to activate the sensor chip surface, followed by immobilization of the recombinant BDNF pro-peptide and blocking of unreacted sites with 1 M ethanolamine (pH 8.5). Immobilization was conducted at 25 °C using approximately 10 µg/mL protein solution in 10 mM sodium acetate (pH 5.0). All BIAcore measurements were carried out at 20 °C with a flow rate of 20 µL/min. Running buffer was HEPES-buffered Saline-EP (HBS-EP) buffer (0.01 M HEPES (pH 7.4), 0.15 M NaCl, 3 mM EDTA and 0.005% Surfactant P20; GE Healthcare). In all BIAcore assays, recombinant neurotrophins and other proteins were diluted to the indicated concentrations with HBS-EP buffer and applied into the BIAcore chamber. In the experiments of Figure 2c, HBS-EP buffer was adjusted to pH 6.5 or 7.0 with HCl solution and using pH meter before binding assays. After binding assays, we confirmed that the buffer pH in BIAcore chamber was as same as that before the assays. *K*_D_, *k*_a_ and *k*_d_ were calculated using the BIAevaluation software, version 3.2 (GE Healthcare). To remove bound proteins, the sensor chip was regenerated with 80 µL of 50 mM NaOH/1 M NaCl.

### 4.4. In Vitro Binding of BDNF and Its Pro-Peptide

BDNF and/or its pro-peptide (100 ng/mL) were incubated in a tube at 4 °C for 1 h. The buffer solution contained 10 mM HEPES (pH 7.4), 150 mM NaCl, 3 mM EDTA and 0.005% NP-40. To immunoprecipitate the protein complex, rabbit proBDNF antiserum [31] or monoclonal anti-BDNF antibody (mAb#9) was added, and the mixture was incubated at 4 °C for 1 h. The immunoprecipitates were analyzed by Western blot using a commercial BDNF antibody (Santa Cruz Biotechnology) or proBDNF antibody (Alomone Labs).

### 4.5. Computational Modeling

The BDNF dimer model was re-constructed using the template of the X-ray structure of the BDNF/NT3 heterodimer complex (PDB: 1BND). Our approach to docking BDNF pro-peptides on the surface of mature BDNF dimer models utilized two steps that took into account several levels of structural flexibility and scoring criteria: (1) rigid-body docking of BDNF pro-peptide to identify the binding position; and (2) refinement of the BDNF pro-peptide/mature BDNF dimer model complex by re-docking with greater structural flexibility and Glide score (Schrödinger, LLC). To obtain the initial binding position of an extended BDNF pro-peptide on the surface of a mature BDNF dimer model, we used ZDOCK (ver.2.3) 16 with the option of sampling at 6-degree rotational steps. Using fast Fourier transform, ZDOCK searches for all possible binding orientations of a BDNF pro-peptide along the surface of a mature BDNF dimer model, optimizing desolvation, shape complementarity and electrostatics. To account for structural flexibility in the refinement step, the Glide Induced Fit Docking (IFD) protocol17 (Schrödinger, LLC) was utilized, followed by iteratively combining rigid-receptor docking (Glide) and protein remodeling by side chain searching and minimization (Prime) techniques. Finally, the best model was rescored according to the binding energy that was calculated using Glide.

### 4.6. Hippocampal Slice Preparation

Hippocampal slices were prepared from male C57BL/6J mice (age 3–4 weeks, Nippon SLC, Shizuoka, Japan) or knockout mice. Mice were maintained according to the guidelines of the Nara Institute of Science and Technology, and all experiments were approved by the Institutional Animal Care and Use Committee. Anesthetized animals were transcardially perfused with ice-cold artificial cerebrospinal fluid (ACSF) to drain the blood from and cool the brain. After each animal was decapitated, the whole brain was removed and immersed in ice-cold (4 °C) ACSF bubbled with a mixture of 95% O_2_ and 5% CO_2_. ACSF consisted of the following (in mM): 125 NaCl, 2.6 KCl, 1.3 MgSO_4_·7H_2_O, 1.24 KH_2_PO_4_, 26 NaHCO_3_, 2.4 CaCl_2_ and 10 d-glucose. Hippocampus transverse slices (400 µm thickness) were prepared using a LinearSlicer Pro7 (Dosaka, Kyoto, Japan). The slices were incubated in ACSF at 30 °C for 30 min, and then maintained at room temperature for at least 120 min before experiments. This treatment was implemented because some plasticity-related signaling returns to baseline levels 2–4 h after slice preparation. Each slice was transferred to a recording chamber where it was placed on nylon nets and perfused continuously with oxygenated ACSF at a flow rate of 2 mL/min. The bath temperature was maintained at 28 °C.

### 4.7. Electrophysiology

For electrophysiology, a glass microelectrode (Narishige, Tokyo, Japan) filled with artificial cerebrospinal fluid (ACSF) (2–4 MΩ electrical resistance) was used to record fEPSPs in the stratum radiatum of area CA1. Experiments were performed as previously described [15]. Extracellular stimulation of the Schaffer collateral pathway was accomplished with a nickel–chromium bipolar stimulating electrode (40 µm diameter; Unique Medical, Tokyo, Japan) placed on either side of the CA3 single-recording electrode in the stratum radiatum. Evoked fEPSPs were amplified (ER-1 amplifier; Cygnus Technology, Inc., Delaware Water Gap, PA, USA) and digitized (DigiData 1200 Interface; Molecular Devices, Palo Alto, CA, USA). Test-stimulus intensity was adjusted to produce baseline fEPSP sizes that were 50% of the maximum evoked fEPSP amplitude, using an SIU-91 constant current isolator (Cygnus Technology, Inc.). Test stimuli were delivered once per minute (0.1 ms stimulus duration) to the Schaffer collaterals. We induced long-term depression (LTD) with single pulses applied at a rate of 1 Hz for 15 min. To prevent nonspecific binding to the equipment (e.g., tubing, slice chamber), the recombinant proteins were dissolved in ACSF containing 1 µg/ml bovine serum albumin (BSA), and then perfused into the chamber.

### 4.8. Statistics

Data are presented as means ± S.E.M. For statistical analyses of the electrophysiological studies, Student’s *t*-test and one-way ANOVA with Tukey’s post-hoc multiple comparison test were used. A *p* value < 0.05 was considered significant.

## 5. Conclusions

Compared with accumulated knowledge of the expression and functions of BDNF, the role of post-translational mechanisms is much less understood. In the present study, we focused on the BDNF pro-domain (pro-peptide), which is generated by proteolytic processing of the precursor BDNF, and first showed that BDNF bound its pro-peptide with high affinity, while other neurotrophins showed no or quite weak binding to the pro-peptide. Secondly, the interaction of BDNF and its pro-peptide was strengthened by a common BDNF polymorphism Val66Met, which replaces valine to methionine in the pro-peptide. Thirdly, the interaction of BDNF and its pro-peptide was more stable in acidic rather than neutral pH conditions. More interestingly, the Val66Met polymorphism increased this binding stability across the entire acidic to neutral pH range. Together, these findings suggest a new role for the BDNF pro-peptide, and provide insight into the common BDNF Val66Met polymorphism, which affects brain functions and is associated with brain diseases.

## Figures and Tables

**Table 1:** Impact of the Val66Met polymorphism on the rate of association and dissociation between BDNF and its pro-peptide. The rate of association (*k*_a_) and dissociation (*k*_d_) and the dissociation constant (*K*_D_) were determined using BIA evaluation 3.2 software (GE Healthcare, Little Chalfont, UK). Note that the mutant Met-containing BDNF pro-peptide decreases the run-on and run-off rates by over 10- and 100-fold, respectively, compared with the wild-type (Val) pro-peptide.

Type of Pro-Peptide	*k*_a_ (1/Ms)	*k*_d_ (1/s)	*K*_D_ (M)
Val-pro-peptide	3.78 × 10^5^	1.59 × 10^−2^	4.21 × 10^−8^
Met-pro-peptide	3.63 × 10^4^	4.44 × 10^−4^	1.22 × 10^−8^
